# Inflammatory Response Mechanisms Exacerbating Hypoxemia in Coexistent Pulmonary Fibrosis and Sleep Apnea

**DOI:** 10.1155/2015/510105

**Published:** 2015-04-05

**Authors:** Ayodeji Adegunsoye, Jay Balachandran

**Affiliations:** Section of Pulmonary & Critical Care, Department of Medicine, University of Chicago, Chicago, IL 60637, USA

## Abstract

Mediators of inflammation, oxidative stress, and chemoattractants drive the hypoxemic mechanisms that accompany pulmonary fibrosis. Patients with idiopathic pulmonary fibrosis commonly have obstructive sleep apnea, which potentiates the hypoxic stimuli for oxidative stress, culminating in systemic inflammation and generalized vascular endothelial damage. Comorbidities like pulmonary hypertension, obesity, gastroesophageal reflux disease, and hypoxic pulmonary vasoconstriction contribute to chronic hypoxemia leading to the release of proinflammatory cytokines that may propagate clinical deterioration and alter the pulmonary fibrotic pathway. Tissue inhibitor of metalloproteinase (TIMP-1), interleukin- (IL-) 1*α*, cytokine-induced neutrophil chemoattractant (CINC-1, CINC-2*α*/*β*), lipopolysaccharide induced CXC chemokine (LIX), monokine induced by gamma interferon (MIG-1), macrophage inflammatory protein- (MIP-) 1*α*, MIP-3*α*, and nuclear factor- (NF-) *κ*B appear to mediate disease progression. Adipocytes may induce hypoxia inducible factor (HIF) 1*α* production; GERD is associated with increased levels of lactate dehydrogenase (LDH), alkaline phosphatase (ALP), and tumor necrosis factor alpha (TNF-*α*); pulmonary artery myocytes often exhibit increased cytosolic free Ca2+. Protein kinase C (PKC) mediated upregulation of TNF-*α* and IL-1*β* also occurs in the pulmonary arteries. Increased understanding of the inflammatory mechanisms driving hypoxemia in pulmonary fibrosis and obstructive sleep apnea may potentiate the identification of appropriate therapeutic targets for developing effective therapies.

## 1. Introduction

Pulmonary fibrosis results from an exaggerated and persistent deposition of extracellular matrix within the lung parenchyma with severe consequences on respiratory function. This process, which is usually irreversible, results in impairment of gas exchange often culminating in significant hypoxemia [1]. The pathophysiologic dysregulatory processes that result in homeostatic alteration involve a complex interplay between inflammation, oxidative stress, chemoattractant mediators, and abnormalities of coagulation, with key cytokines driving the underlying proinflammatory and profibrotic mechanisms [1]. The abnormal accumulation of scar tissue within the lung parenchyma, which characterizes fibrotic lung diseases, can be seen in several interstitial lung diseases including idiopathic pulmonary fibrosis (IPF), connective tissue disease associated interstitial lung disease (CTD-ILD), and chronic hypersensitivity pneumonitis. Other pulmonary diseases like sarcoidosis, combined pulmonary fibrosis and emphysema (CPFE), and cryptogenic organizing pneumonia (COP) also exhibit parenchymal lung fibrosis to varying extents. IPF, however, is the most common idiopathic ILD [2] and carries the greatest severity amongst these etiologies of pulmonary fibrosis with multiple studies performed to elucidate the underlying fibrotic mechanisms involved. IPF is a progressive and uniformly fatal pulmonary fibrosis of unknown cause affecting over 128,000 people in the United States [3]. Its current median survival from diagnosis is 2-3 years and its global mortality is rising [4]. The irreversible fibrosis in IPF has been associated with abnormal signaling pathways involving the production of proinflammatory and profibrotic cytokines [5–7].

Obstructive sleep apnea (OSA) is remarkably common in patients with IPF for unclear reasons [8]. While 2–4% of healthy adults have OSA syndrome [9], several recent studies report a prevalence as high as 88% in patients with IPF [8, 10, 11]. Appropriate use of CPAP in patients with OSA reduces nocturnal desaturations and the detrimental effects of systemic hypoxemia. Thus effective treatment of OSA has been recognized as one of the cornerstones of management in these patients and improves mortality [12].

While patients with IPF are already predisposed to severe hypoxemia, the sequence of desaturation-reoxygenation that characterizes intermittent hypoxemia in OSA constitutes a more potent stimulus than continuous hypoxia for oxidative stress and culminates in systemic inflammation and generalized vascular endothelial damage [13, 14]. This sleep-associated intermittent oxygen desaturation exceeds the hypoxemic episodes which occur during maximal exercise and negatively impacts survival in patients with IPF [15].

### 1.1. Clinical Entities Exacerbating Hypoxemia in Fibrotic Lung Disease

**Figure 1:**
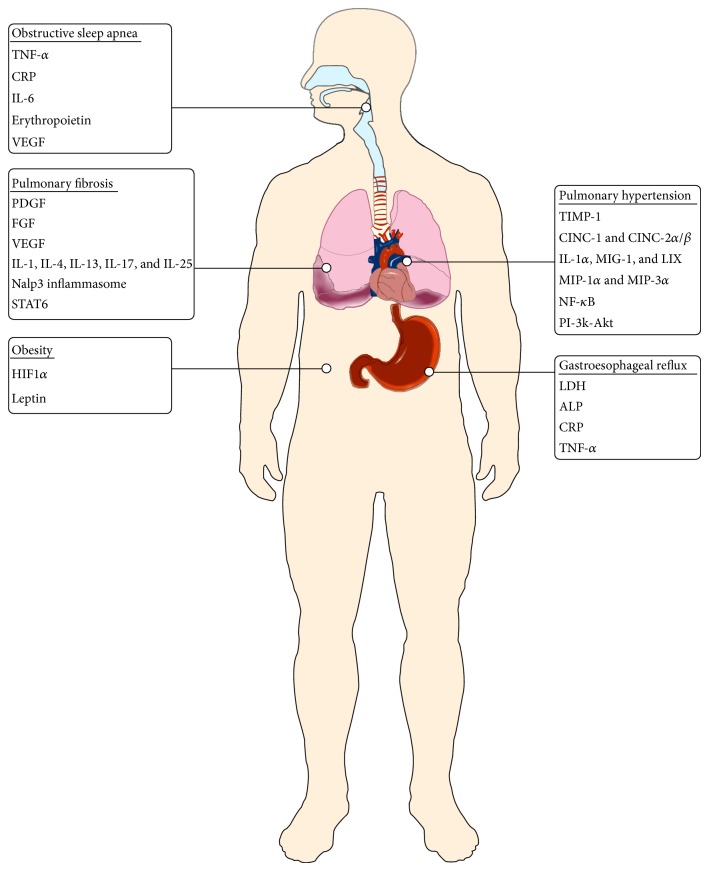
Inflammatory mediators of hypoxemia in coexistent pulmonary fibrosis and sleep apnea.

Patients with pulmonary fibrosis frequently experience acute exacerbations that aggravate the underlying hypoxemia and worsen outcomes [16, 17]. While the underlying etiology in many cases remains idiopathic or attributed to infective causes, it is believed that a significant proportion is attributable to the presence of chronic comorbid conditions frequently observed in fibrotic lung diseases [17] (Figure 1). Often, these chronic conditions which are also present in patients with OSA contribute to the amplification of hypoxemia by the release of chemoattractants, hormones, and specific inflammatory mediators which alter the fibrotic pathway within the pulmonary parenchyma. The coexistence of moderate to severe OSA results in episodes of hypoxic stress which directly modulates expression of cell adhesion molecules responsible for mediating leukocyte adhesion to endothelial cells [18]. Repetitive hypoxemia in OSA patients with fibrotic lung diseases ultimately leads to significantly increased levels of these cell adhesion molecules.

(1) Pulmonary hypertension (PH) is a pathophysiologic condition which frequently occurs in fibrotic lung diseases. When associated primarily with chronic respiratory disorders, it is classified as WHO Group 3, mild to moderate precapillary PH which worsens the prognosis of the underlying pulmonary disease. However, other WHO groups of PH (pulmonary arterial hypertension, chronic thromoembolic PH) also occur in these patients and knowledge regarding the various inflammatory and pathologic processes which result in progressive microvascular injury remains limited [19, 20]. Recognition of PH out of proportion with the underlying chronic respiratory disease carries management and prognostic implications. Various inflammatory cytokines play key roles in progression of PH; these include tissue inhibitor of metalloproteinase (TIMP-1), cytokine-induced neutrophil chemoattractant (CINC-1, CINC-2*α*/*β*), interleukin- (IL-) 1*α*, monokine induced by gamma interferon (MIG-1), lipopolysaccharide induced CXC chemokine (LIX), macrophage inflammatory protein- (MIP-) 1*α*, MIP-3*α*, and nuclear factor- (NF-) *κ*B [20].

(2) Obesity, often associated with dysregulation of metabolic pathways, is frequently present in patients with OSA, IPF, or both. Adipocytes produce increased levels of leptin, a hormone responsible for regulating appetite suppression but which may induce platelet regulation and occurs in OSA at much higher levels [18]. Effective treatment of OSA has been demonstrated to reduce the acumulation of visceral fat and predisposition to weight gain [21–23]. Furthermore, adipocyte induction of hypoxia inducible factor 1*α* (HIF1*α*) is critical to the fibrotic response and has been linked directly to progression of metabolic dysfunction in the presence of hypoxia [24].

(3) Gastroesophageal reflux disease (GERD) which commonly results in occult microaspiration of acidic gastric contents is exceedingly common in patients with IPF similar to its high prevalence in OSA. Frequently, all three conditions occur concurrently and whether GERD plays a key role in the onset and progression of IPF or results from morphologic and mechanistic alteration of the diaphragmatic esophageal hiatus following lung fibrosis remains unknown [25]. Recent studies have also shown a significant increase in the bronchoalveolar fluid levels of several inflammatory biomarkers (lactate dehydrogenase (LDH), alkaline phosphatase (ALP), and tumor necrosis factor alpha (TNF-*α*)) in patients with GERD and IPF when compared to those without IPF [26]. Serum samples of IPF patients with GERD analyzed for LDH, ALP, and CRP also demonstrated elevated levels.

(4) Hypoxic pulmonary vasoconstriction (HPV) occurs as a compensatory physiological mechanism to optimize ventilation-perfusion matching and maintain efficient gas exchange by shunting blood from areas of the lungs that are poorly ventilated to the better ventilated areas. When hypoxia is persistent or chronic as observed in pulmonary fibrosis or OSA, sustained pulmonary vasoconstriction and medial hypertrophy of the pulmonary vasculature result in hypoxia-induced pulmonary hypertension (HPH). Chronic hypoxia increases the resting levels of cytosolic free Ca2+ in pulmonary arterial smooth muscle cells, thus acting as a major stimulus for cell proliferation and migration [27]. Proinflammatory cytokines locally produced in the pulmonary arterial tissue are thought to be upregulated and expressed under hypoxic conditions such as what occurs in patients with IPF and OSA. Hypoxemia alone has been shown to be the most potent stimulus for the release of these proinflammatory cytokines by macrophages. Pulmonary arterial expression of TNF-*α* and IL-1*β* has been demonstrated under hypoxic conditions and the observed upregulation of these cytokines is dependent on the activation of protein kinase C (PKC) [28].

## 2. Sleep Disorders and Sleep-Related Breathing Disorders in Pulmonary Fibrosis

Sleep disorders, sleep fragmentation, and sleep-related breathing disorders occur commonly in IPF. This may be attributable to the increased prevalence of nocturnal oxygen desaturations, coughing with arousals, and increased respiratory drive that occur in these individuals. The International Classification of Sleep Disorders (ICSD) categorizes sleep-related breathing disorders in idiopathic pulmonary fibrosis to a separate group termed “sleep disorders with sleep-related hypoventilation and hypoxemia in parenchymal or vascular lung diseases” [29] and the most recent ICSD-3 classification in 2014 placed them in the unique subgroup of “sleep-related hypoxemia disorder” [30] given the distinctive nature of oxygen desaturation that occurs in these individuals. The nocturnal pulse oximetry curve of a typical patient with severe IPF usually follows a specific pattern; other than a low oxygen saturation nadir at night, multiple phasic desaturations frequently occur from hypoventilation, which may lead to fragmentation of sleep and impairment of sleep quality [31]. Furthermore, a reduction in REM sleep and an increase in Stage 1 sleep have been well documented in patients with severe pulmonary fibrosis [32–34]. Daytime PaO_2_ levels while awake appear to be predictive of the extent of nocturnal oxygen desaturation and presence of OSA in IPF patients during sleep ultimately leads to increasingly profound oxygen desaturations exceeding that present in individuals with isolated sleep apnea [31].

### 2.1. Mechanisms of Sleep-Related Hypoxemia and Hypoventilation

Though multiple significant oxygen desaturations are characteristic of nocturnal oximetry in persons with sleep apnea, the physiologic mechanism driving sleep disordered breathing in the presence of pulmonary fibrosis manifests predominantly as hypopneas rather than apneic events [8, 35]. Interestingly a recent study showed that pulmonary function parameters such as lung volumes, FVC, and DLCO do not correlate inversely with the Apnea-Hypopnea Index (AHI) as would be expected [8]. However, like patients with isolated OSA, daytime sleepiness as measured by the Epworth Sleepiness Scale is a poor predictor of OSA severity. The observed nocturnal hypoxia almost invariably disrupts sleep architecture and appears to have a greater impact on the quality of life in these individuals [35, 36]. In a small cohort of patients AHI appeared to correlate positively with BMI and negatively with FEV1 [34]. The impairment in pulmonary physiology appears to be linked to the interdependence between upper airways in OSA and lung volumes. In snorers with OSA, a decrease in lung volumes results in a dramatic reduction in the pharyngeal cross-sectional area, a phenomenon that is drastically amplified by the fibrotic process which occurs in individuals with diffuse parenchymal lung fibrosis. Studies in which the functional residual capacity was increased have depicted a subsequent improvement in sleep architecture and improvement in AHI [37, 38]. Recurrent, chronic microaspiration associated with GERD worsens hypoxemia in patients with OSA and IPF; studies have shown amelioration of GERD when the OSA in these patients is treated with CPAP, which could potentially lead to a retardation of disease progression [39].

### 2.2. Inflammatory Role of Sleep-Disordered Breathing in Fibrotic Lung Disease

Episodic intermittent hypoxemia in sleep-disordered breathing stimulates occurrence of significant oxidative stress, systemic vascular endothelial damage, and inflammation [13, 14]. This results in the generation of highly reactive superoxide radicals, which facilitates reperfusion-mediated endothelial injury. Free oxygen radicals released from polymorphonuclear leukocytes during hypoxemic episodes further propagate this vascular oxidative stress. Occurrence of episodic hypoxemia from nocturnal desaturations also activates carotid chemoreceptors, thereby triggering arteriolar vasoconstriction and systemic catecholamine secretion [18].

Serum levels of tumor necrosis factor-*α*, interleukin-6, and C-reactive protein (CRP) may also be elevated in response to sleep deprivation and hypoxia, both of which occur in OSA and in IPF. CRP mediates the inhibition of nitric oxide synthase, thus leading to an increase in the expression of certain cell adhesion molecules thereby modulating the adhesion of leukocytes to endothelial cells. This may be responsible for elevated levels of cell adhesion molecules in individuals with fibrotic lung disease who experience severe OSA [40–48].

## 3. Chemoattractants and Inflammatory Mediators of Pulmonary Hypertension (PH)

Pulmonary hypertension is characterized by an increase in the pulmonary vascular resistance resulting from structural alteration to the microcirculation and eventual obstructive proliferative changes. Multiple inflammatory processes precede this pulmonary arteriolar remodeling. Endothelial dysfunction, medial hypertrophy from microvascular injury, adventitial thickening, perivascular inflammatory infiltrates, and reactive oxygen species all contribute to the remodeling process that culminates in progression of PH [49–51]. Proinflammatory mediators produced by the pulmonary epithelium, infiltrating macrophages, and lymphocytes as well as locally expressed chemokines abound within the plexiform lesions that characterize PH [52, 53]. Levels of macrophage inflammatory protein- (MIP-) 1*α*, MIP-3*α*, interleukin- (IL-) 1*α*, and tissue inhibitor of metalloproteinase (TIMP-1) are elevated within the lungs in murine models of PH. Similarly, patients with PH exhibit a demonstrable increase in circulating levels of IL-1*β*, IL-6, P-selectin, and macrophage inflammatory protein-1*α*. Patients with severe PH also demonstrate an increase in inflammatory cell infiltrates associated with an enhanced pulmonary expression of fractalkine and other chemokines, confirming the crucial role played by inflammation in PH progression.

Macrophages release MIP chemokines which activate lymphocytes and granulocytes, thereby enhancing the synthesis of TNF-*α*, IL-1, IL-6, and other proinflammatory cytokines vital to the pathogenesis of PH. Histopathologic specimens of patients with PH demonstrate increased MIP-1*α* mRNA, and serum samples show increases in IL-1 and IL-6. Activated macrophages and neutrophils as well as various epithelial and endothelial cells produce these cytokines which are responsible for initiation and progression of inflammatory responses and oxidative stress [54–57].

Fibroblasts in PH express mitogen-activated protein kinases (MAPKs), a class of central signaling molecules, which respond to various stimuli by phosphorylating diverse substrates such as transcription factors and other kinases. These intricate processes result in the orchestration of cell survival, apoptosis, proliferation, differentiation, and other inflammatory measures associated with pulmonary vascular remodeling. The MAPK pathway has also been associated with many of the inflammatory mediators that characterize PH [58–61].

NF-*κ*B regulates the expression of these cytokines and chemokines via the activation of the associated genes in patients with PH. The NF-*κ*B inhibitor, I-*κ*B that may be activated by signaling kinases like MAPKs, facilitates the retention of NF-*κ*B in the cytoplasm as an inactive form. Removal of I-*κ*B unmasks the nuclear localization signal for NF-*κ*B and its subsequent translocation to the nucleus where phosphorylation and acetylation result in NF-*κ*B dependent gene expression [62–64]. NF-*κ*B also plays a key role in regulating the expression of matrix metalloproteinase, thereby indirectly influencing an increase in TIMP-1 expression [20, 65].

Phosphatidylinositol 3-kinase-Akt (PI-3k-Akt) signaling is also essential to the regulation of cell proliferation, migration, and survival. PI-3k and Akt proteins can be found in vascular smooth muscle cell (VSMC) cultures where they are constitutively expressed; whereas vascular remodeling following injury and VSMC proliferation is characterized by an increase in activated Akt and smooth muscle specific gene expression. Endothelial nitric oxide synthase (eNOS) is activated by the PI-3k-Akt pathway and promotes VSMC relaxation. Conversely, the PI-3k-Akt pathway inactivates glycogen synthase kinase (GSK)-3*β*, which indirectly prevents necrotic cell death [66–69].

### 3.1. Pulmonary Hypertension in Fibrotic Lung Disease

The occurrence of PH in fibrotic lung diseases such as IPF is well recognized and its presence or absence in IPF significantly impacts survival. The presence of pulmonary vascular bed destruction and diffuse fibro-proliferative phenomena that characterize these patients may account for the observed increase in mortality. These patients however, exhibit a weak correlation of their FVC with severity of PH; patients may thus demonstrate severe PH in the presence of relatively preserved lung volumes. This increased severity of PH that is not fully explained by the extent of parenchymal fibrosis has been termed “out of proportion PH”; replacing the previously vague term—“pulmonary heart”. These patients are typically identified when the pulmonary artery pressure exceeds PAPm > 35 mmHg; the increased pressures are believed to result from extravascular pulmonary parenchymal disease and not from diseased pulmonary arteries. However, idiopathic pulmonary arterial hypertension may also occur in patients with fibrotic lung diseases [19, 70, 71].

### 3.2. Pulmonary Hypertension in Obstructive Sleep Apnea (OSA)

Up to 42% of patients with OSA exhibit PH; PaO_2_, PaCO_2_ and FEV1 appear to be significant determinants in the pathogenesis of PH in this population. Multiple transcription factors such as hypoxia-inducible factor 1 (HIF-1) play key roles in this process and are more strongly activated by intermittent hypoxia than continuous hypoxia. Cytokines and growth factors such as erythropoietin and vascular endothelial growth factor are also activated and act in conjunction with endothelin-1 (ET-1) in modulating pulmonary artery structure and pressure. Non-invasive ventilation has been shown to improve levels of pro-BNP, severity of PH and baseline exercise capacity in these patients [72–75].

### 3.3. Pulmonary Hypertension in Obesity Hypoventilation Syndrome

The majority of individuals with morbid obesity maintain eucapnia despite the significant load placed on the respiratory system by obesity. Physiologic compensatory mechanisms account for the impairment in respiratory muscle function, ventilatory control and additional stress on the work of breathing. A fraction of these patients will however lapse into a chronic pattern of daytime hypercapnia and develop the obesity hypoventilation syndrome. The features of this syndrome include a BMI ≥ 30 kg/m^2^, chronic alveolar hypoventilation, daytime hypercapnia (PaCO_2_ ≥ 45 mmHg) and either sleep respiratory disorders or hypoventilation during sleep. Up to 88% of patients with OHS have simultaneous PH and while it is often unrecognized, this condition carries a significantly increased mortality risk in comparison to patients with sleep-disordered breathing who are eucapnic [76].

## 4. Hypoxia Signaling in Pulmonary Hypertension

Hypoxic conditions stabilize transcription factors such as hypoxia inducible factors (HIFs) to promote adaptation to hypoxia. They are also instrumental in regulating the release of erythropoietin under hypoxic or anemic states to enhance erythropoiesis and modulate the underlying processes in ischemic and inflammatory diseases [77–87].

### 4.1. The Role of Hypoxia Inducible Factors

HIFs are central to the regulation of stress, metabolism and tissue adaptation to the diminution in oxygen availability. These heterodimeric transcription factors regulate oxygen homeostasis globally and are stabilized in hypoxia when the oxygen dependent degradation domain of the *α*-subunit forms a functional complex with its corresponding *β*-subunit. Reversal of hypoxia promotes rapid degradation of HIFs. Three isoforms are commonly implicated in cellular oxygen-sensing and signaling: HIF1*α*—purified through its association with the EPO gene; HIF2*α*—with heterodimeric links to HIF1*β*; and HIF3*α*—a distant isoform that inhibits gene induction dependent on hypoxia response element (HRE). Prolyl hydroxylases (PHDs) produced in mammalian cells target HIF1*α* and HIF2*α* via a proteasomal pathway dependent on E3 ubiquitin ligase for their degradation, thereby regulating HIF activity [87–94].

### 4.2. Interdependence of Hypoxia and Inflammation

Hypoxia and ischemia characterize and drive a broad range of inflammatory conditions. Inflammatory lesions frequently demonstrate the occurrence of tissue hypoxia that may result from concurrently increased tissue metabolism and reduced oxygen supply, a condition commonly referred to as inflammatory hypoxia. Increased vascular resistance, occlusion and thrombosis typical of PH could lead to dramatic shifts in the metabolic demand and supply ratios thereby result in pulmonary inflammatory hypoxia. While this hypoxic effect may lead to the stabilization of HIFs, other pro-inflammatory molecules such as lipopolysaccharides, toll-like receptors signals and metabolic by-products like succinate could also result in similar effects [85, 86, 95–100].

Also, hypoxia by itself causes tissue edema and breakdown and acts as an inflammatory stimulus to increase cytokine levels. Several studies have depicted a diminution in the tissue barrier function and simultaneously increased vascular leakage in hypoxia-driven inflammation, providing further insights into the underlying mechanisms of hypoxia in inflammatory diseases. Though, NF-*κ*B signaling activates the transcription of HIF1*α*, it appears HIF1 also regulates the hypoxia-associated increase in NF-*κ*B activity in a pathway that includes Notch and activating transcription factor 4. The anti-inflammatory functions of the HIFs and NF-*κ*B appear to be regulated via hydroxylase inhibitors, which hydroxylate components of the interleukin-1*β* (IL-1*β*) pathway [101, 102].

## 5. Mediation of Lung Inflammation and Fibrosis by Hypoxia-Induced Mitogenic Factor (HIMF)

Hypoxia-induced mitogenic factor (HIMF), a cysteine rich protein which exhibits chemotactic and mitogenic properties during the vascular remodeling associated with PH has also been described to exhibit fibrogenic properties in pulmonary fibrosis. Pulmonary expression of these proteins may be regulated by cytokines classically associated with Th2 pathway such as IL4 and IL13. Murine models of hypoxia-induced PH however suggest that the triggers for HIMF do not necessarily require Th2 regulation. The production of chemokines and angiogenic factors such as stromal-derived factor-1, vascular endothelial growth factor and MCP-1 appear to specifically depend on the IL-4 signaling system. HIMF promotes fibrosis in an IL-4 dependent fashion by inducing differentiation of myofibroblasts and increasing the expression of *α*-SMA as well as type I collagen thereby promoting collagen accumulation within pulmonary tissues [103–107].

### 5.1. Pro-Inflammatory and Pro-Fibrotic Cytokines Contributing to Fibrosis

IPF is currently believed to occur following the abnormal proliferation and remodeling of fibrous tissue within the lung parenchyma as a result of dysfunctional signaling pathways involving alveolar epithelial cells and pulmonary fibroblasts. Tyrosine kinase mediated pathways involving platelet-derived growth factor (PDGF), fibroblast growth factor (FGF) and vascular endothelial growth factor (VEGF) are thought to be abnormally activated in these patients [6, 108–110].

IL-1, which is associated with acute lung injury, may also contribute to the pathogenesis of pulmonary fibrosis. IL-1 induced fibrosis has been demonstrated to be associated with an increase in TNF expression suggesting a mechanistic association. Lung biopsy samples and serum from patients with IPF exhibit increased levels of TNF. Furthermore, IL-1 increases the levels of pro-fibrotic cytokines such as TGF-*β*1 and platelet-derived growth factor (PDGF) [111–114].

There is increasing evidence pointing to key roles played by T cells in both the inflammatory and maintenance phases of pulmonary fibrosis. CD4^+^ Th1 and Th2 cells and their cytokines appear crucial to the pathogenesis of pulmonary fibrosis. Upon exposure to bleomycin, CD4^+^ T cells demonstrate significant production of IL-17A, a cytokine implicated in the pathogenesis and progression of pulmonary fibrosis [115–118]. Th2 cytokines (IL-4 and IL-13) drive the expression of HIMF found in inflammatory zone 1 (FIZZ1), a marker for those M2 macrophages activated via an alternative pathway, in the early phases of murine hypoxic models. Resistin-like molecule *β* (RELM*β*), the human correlatione of HIMF has also been observed to be elevated in the lungs of individuals with PH and lung fibrosis from scleroderma [119–122].

Oxidative stress, mediated by the Nalp3 inflammasome, reactive oxygen species (ROS) and NADPH oxidase (NOX) activity, is known to perpetuate pulmonary pro-fibrotic inflammatory responses [123–126]. While IFN-*γ* is known to inhibit fibrosis, IL-5 may promote pulmonary fibrosis by its effect on recruiting eosinophils and the subsequent production of TGF-*β*1, PDGF, and IL-13. IL-25 also appears to play a key role in the progression of pulmonary fibrosis in patients with IPF [127–131].

### 5.2. STAT6 and Pulmonary Inflammation

Regulation of inflammatory responses within the lungs has also been shown to involve signal transducer and activator of transcription 6 (STAT6), which plays a key role in modulating smooth muscle changes, B cell IgE production, airway eosinophilia, Th2 cell differentiation and epithelial mucus production in animal models [132]. STAT6 also lies downstream of IL-4 and IL-13, which exhibit increased levels in human airway inflammatory diseases and are believed to play key roles in progression of pulmonary fibrosis. IL-4 and IL-13 stimulate fibroblast differentiation and expression of collagen thus contributing to airway fibrosis in interstitial lung disease [132, 133]. Overexpression of the IL-13 transgene induces persistent smooth muscle hypertrophy and subepithelial fibrosis; an effect partly mediated by STAT6. Patients with pulmonary fibrosis from chronic hypersensitivity pneumonitis exhibit a predominantly Th2 pattern within the interstitial compartment of their lungs characterized by IL-4 and IL-5 activity. Also, individuals with pathologic features of IPF demonstrated significant elevations in the expression of gene and protein levels of IL-4R*α*, IL-13R*α*1 and IL-13R*α*2 [129, 132, 134, 135].

## 6. Inflammatory Correlates of Obesity and Gastroesophageal Reflux

Fibrotic lung diseases are known to frequently coexist with obesity, GERD and chronic occult microaspiration especially in the presence of sleep related breathing disorders [136, 137].

Metabolic dysregulation linked to sleep apnea may also increase the tendency for weight gain and the majority of IPF patients with OSA are commonly observed to have a significantly increased BMI [8, 18]. The ensuing inflammatory responses emanating from co-existence of obesity and GERD in patients with sleep related breathing disorders might perpetuate the occurrence and progression of fibrosis within the lungs.

### 6.1. Obesity-Related Inflammatory Mediators of the Fibrotic Response

The underdeveloped vascular system within the expanded adipose tissue of patients with obesity predisposes adipocytes to oxygen deficits, and continued overnutrition rapidly leads to a state of chronic persistent hypoxia within the adipose tissue. Protracted high levels of hypoxia have been demonstrated within the white adipose tissue of obese individuals resulting in the induction of HIF-1 as an adaptive response [24, 138–143].

In contrast to the function of HIF-1 in other tissues, excessive levels do not initiate a proangiogenic response within adipocytes but rather induces a transcriptional pathway that leads to the production of extracellular matrix components (ECM) and eventual deposition of extensive fibrous tissue. Further infiltration by inflammatory cells results in the formation of dysfunctional adipose tissue and a metabolic profile that is ultimately unfavorable. Increased production of leptin by adipocytes is also observed in patients with OSA at levels exceeding those exhibited by obese patients with no sleep related breathing disorders. These elevated levels may portend increased cardiovascular risk [144–146].

### 6.2. Gastroesophageal Reflux Disease in Pulmonary Fibrosis and Sleep Apnea

The triad of fibrotic lung disease, OSA and GERD is now recognized to occur quite frequently especially in patients with IPF and multiple studies suggest a possible under-recognition of OSA in fibrotic lung disease [25]. The distal bronchi of patients with a recent diagnosis of IPF have a more acidic milieu than those in patients with a new diagnosis of GERD and no fibrotic lung disease [26]. While it is thought that increased transdiaphragmatic pressure gradients, altered pulmonary mechanics and occurrence of hiatal hernia may contribute to this observation, chronic inflammation within the lungs of patients with IPF may play an important role due to the presence of local acidity and H^+^ elevation that accompanies cellular injury, apoptosis and tissue necrosis [147–151]. Elevation of LDH, a marker of cell injury was noted to be almost three times higher in the bronchoalveolar fluid of IPF patients than in their serum. Similarly, a five-fold elevation in bronchoalveolar LDH levels is observed in patients with IPF when compared to individuals with GERD suggestive of an active local inflammatory process.

TNF-*α*, which stimulates fibroblasts and facilitates collagen production, and ALP, a tissue biomarker of cellular damage, have been demonstrated to be elevated in the lungs of patients with IPF [26, 152]. Individuals with IPF also demonstrate an increase in the population of type 2 innate lymphoid cells (ILC2) within their lungs implying that they play a key role in pulmonary inflammation and fibrosis [131].

Recent studies reveal an increased bacterial burden within the lungs of IPF patients that is predictive of a decline in lung function [153, 154]. Interestingly, this increase in the pulmonary bacterial burden may contribute to the ongoing chronic inflammatory process. However, whether this increase in pulmonary bacterial flora is related to chronic occult microaspiration remains yet to be determined.

### 6.3. Corticosteroids and Their Effects

The use of corticosteroids confers no benefit on improving pulmonary function or mortality in patients with IPF [155–157] and when they inhibit endogenous suppressive pathways they may be harmful [158]. Though they remain commonly used for acute exacerbations of fibrotic lung disease, their use may elevate the risk for sleep related breathing disorders by causing obesity [11].

Of importance is the realization that corticosteroid resistance does not necessarily imply an absence of inflammatory involvement, as several inflammatory diseases are known to respond poorly to conventional anti-inflammatory therapies [157, 159].

## 7. Effects of Current Anti-Inflammatory and Anti-Fibrotic Therapies in Hypoxia

Advances in new therapies focused on the signaling pathways of abnormal wound healing in response to epithelial injury have resulted in recent approval of pirfenidone and nintedanib for the treatment of patients with IPF.

Pirfenidone is an oral antifibrotic drug shown to reduce disease progression and decline in lung function in patients with IPF [5]. It suppresses the production of TNF*α*, IL-1 and IL-6 while stimulating the production of IL-10, an anti-inflammatory cytokine [160]. Its antifibrotic effect is mediated by attenuating the induction of alpha-smooth muscle actin by TGF-*β* and reduction of fibroblast proliferation. Other key factors in this pathway such as Akt, p38 and Smad3 are also inhibited by pirfenidone [161]. CX3CL1 (fractalkine) and its receptor (CX3CR1) are both strongly induced by TNF*α*. In human placental models of inflammation and hypoxia, pirfenidone non-selectively inhibits TNF*α* and other inflammatory mediators resulting in a reduction of the up-regulation of CX3CR1, which is necessary for a subsequent increase in CX3CL1 production in hypoxic conditions [162]. Though fibrotic lung diseases result in a deficiency of oxygen at the cellular level, reactive oxygen species (ROS) are paradoxically responsible for a significant amount of tissue damage in these conditions [163]. The fibroblasts of individuals with IPF have been shown to produce ROS such as hydrogen peroxide (H_2_O_2_) when stimulated with TGF-*β* [164]. The anti-oxidant effect of pirfenidone reduces the tissue burden of ROS and the pirfenidone-iron complex exhibits significant scavenging activity against superoxide radicals [165, 166].

Nintedanib is a triple angiokinase intracellular inhibitor of multiple tyrosine kinases including the fibroblast growth factor (FGF), vascular endothelial growth factor (VEGF) and platelet-derived growth factor (PDGF) receptors [6]. In preclinical models of pancreatic and lung cancer nintedanib demonstrated potent anti-angiogenic effects while increasing levels of hypoxia. However, despite this increase in hypoxia, markers that characterize the epithelial to mesenchymal transition that accompanies fibrosis were not elevated [167].

## 8. Summary & Future Perspectives

Fibrotic lung disease and sleep-related breathing disorders frequently co-exist and the hypoxic disease course experienced by these patients is often exacerbated by the presence of co-morbid conditions like obesity, pulmonary hypertension, GERD and chronic occult microaspiration, which perpetuate the underlying inflammatory mechanisms. Identification of appropriate therapeutic targets along the various inflammatory pathways may yield new insights into developing effective treatment of this fatal condition.

Treatment with CPAP has been effective in reducing systemic levels of inflammatory biomarkers in patients with OSA and improving the quality of life in those with concomitant IPF. However, more studies are required to determine if effective amelioration of oxygen desaturation in IPF patients with OSA would confer a survival benefit especially in the context of recently approved therapies for IPF [168].
